# On the shape-dependent propulsion of nano- and microparticles by traveling ultrasound waves

**DOI:** 10.1039/d0na00099j

**Published:** 2020-07-21

**Authors:** Johannes Voß, Raphael Wittkowski

**Affiliations:** Institut für Theoretische Physik, Center for Soft Nanoscience, Westfälische Wilhelms-Universität Münster D-48149 Münster Germany raphael.wittkowski@uni-muenster.de

## Abstract

We address the propulsion mechanism of ultrasound-propelled nano- and microparticles that are exposed to a traveling ultrasound wave. Based on direct computational fluid dynamics simulations, we study the effect of two important aspects of the particle shape on the propulsion: rounded *vs.* pointed and filled *vs.* hollow shapes. We also study the flow field generated around such particles. Our results reveal that pointedness leads to an increase of the propulsion speed, whereas it is not significantly affected by hollowness. Furthermore, we show that the flow field near to ultrasound-propelled particles can look similar to the flow field generated by pusher squirmers.

## Abstract 2

We address the propulsion mechanism of ultrasound-propelled nano- and microparticles that are exposed to a traveling ultrasound wave.

## Graphical abstract

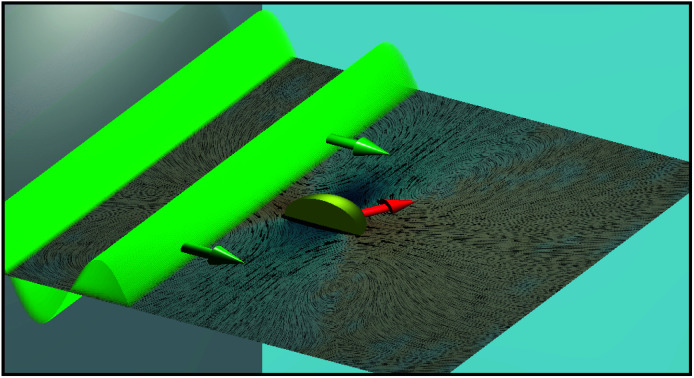

## I. Introduction

With the experimental discovery of fuel-less ultrasound-propelled colloidal particles in 2012,^1^ important potential applications of motile nano- and microparticles (also called “active particles”)^2^ have become within reach.^3^ One of their most attractive potential applications is the usage as propelled nano- or microdevices in medicine^4–6^ that allows for targeted drug delivery,^7–12^ enhanced biodetoxification,^13,14^ nanosurgery,^15,16^ enhanced diagnostics,^4,17–19^ and many more fascinating applications.^16,20–27^ In contrast to different propulsion mechanisms like chemical propulsion,^20,28,29^ other fuel-based propulsion,^30^ light-propulsion,^31^ and X-ray propulsion,^32^ the acoustic propulsion^1,5–9,13,18,33–44^ has important advantages: it is fuel-free, biocompatible, and allows to supply the particles continuously with energy. Similar to chemically powered particles, ultrasound-propelled particles are supplied only with energy but not with directional information so that their direction of motion is determined only by their instantaneous orientations. Ultrasound-propelled particles can be rigid^1,7,9,33–38,41,42,44–47^ or have moveable components.^30,42,48,49^ The latter ones include bubble-propelled particles,^30,49^ which can reach rather high propulsion speeds, but since the former ones are easier to produce, they are more likely to be applied in the near future. There exist also hybrid particles combining acoustic propulsion with a different propulsion mechanism.^37,40,44,50,51^

Ultrasound-propelled nano- and microparticles have been intensively investigated in recent years.^1,33–35,38,39,41,42,44–47,52–54^ While the most studies are based on experiments,^1,33–35,38,39,41,42,44,47^ there are only two theory-based studies so far.^45,46^ Despite the large number of existing studies on acoustically propelled particles, we are still at the beginning of exploring and understanding their features. Even the details of their propulsion mechanism are still unclear. For example, it is not yet known how the propulsion speed depends on the properties of the particles and their environment, what the maximal speed of the particles for a given ultrasound intensity is, and which structure the flow field generated around the particles has. One of the most basic properties of the particles is their shape. Nevertheless, only very few particle shapes have been considered so far. The main reason for this is that the particle shape cannot easily be varied in experimental studies and that the existing theoretical studies focus on the particle shapes used in the experiments. The particle shapes studied so far are mostly cylinders with a concave and a convex end.^1,34–36,47^ As a limiting case, also cup-shaped particles were studied.^38,44^ Apart from that, there exist only a study that addresses gear-shaped particles^41^ and studies on particles with movable components^30,42,48,49^ and thus a nonconstant shape.

For a cylindrical shape with spherical concave and convex ends, the direction of movement was found in experiments to point towards the concave end,^39^ but the newest theoretical study suggests that this depends on the shape of the caps.^46^ The direction of propulsion seems to depend also on the length of the cylinder, since cup-shaped particles were found to move towards their convex end.^38^ Hence, we can conclude that the propulsion speed depends sensitively on the particles' shape, but we have not yet a deeper understanding of this dependence.

A better understanding of the shape-dependence of the propulsion would be helpful for future studies, since it would provide a good opportunity for optimizing the particle speed and thus the efficiency of the particles' propulsion. Large propulsion speeds are crucial for medical applications, where the maximal ultrasound intensity is limited by the requirement of biocompatibility and the particles must be fast enough to withstand the blood flow that tends to carry the particles away. The fastest ultrasound-propelled fuel-free particles observed so far reached a speed of about 250 μm s^−1^.^33^ This is faster than the blood flow in the vascular capillaries with a typical speed of about 100 μm s^−1^,^16^ but the transducer voltage of 10 V applied in the corresponding experiments indicates that the acoustic energy density was about 10–100 J m^−3^ (ref. 55) and thus too high for usage in the human body, where the energy density should be below 4.9 J m^−3^ to avoid damage to the tissue.^56^

A further limitation related to the experiments is the usage of a standing ultrasound wave in all but one^42^ experimental studies. To facilitate observation of the particles with a microscope, they are enclosed by a thin chamber with two parallel horizontal walls, of which at least the upper one is transparent, and a standing wave field that levitates the particles in a nodal plane between the horizontal walls of the chamber. However, in many important potential applications, such as medical ones, the sound waves would be traveling.

In this article, we advance the knowledge about the acoustic propulsion of homogeneous rigid nano- and microparticles that are exposed to traveling ultrasound waves. Using direct computational fluid dynamics simulations based on numerically solving the compressible Navier–Stokes equations, we determine the sound-induced forces acting on these particles together with their resulting propulsion velocity as well as the flow field generated around the particles. To address the shape-dependence of these quantities, we consider some particle shapes that differ with respect to two aspects not studied previously: we compare rounded with pointed shapes and filled with hollow ones.

## II. Methods

**Fig. 1:**
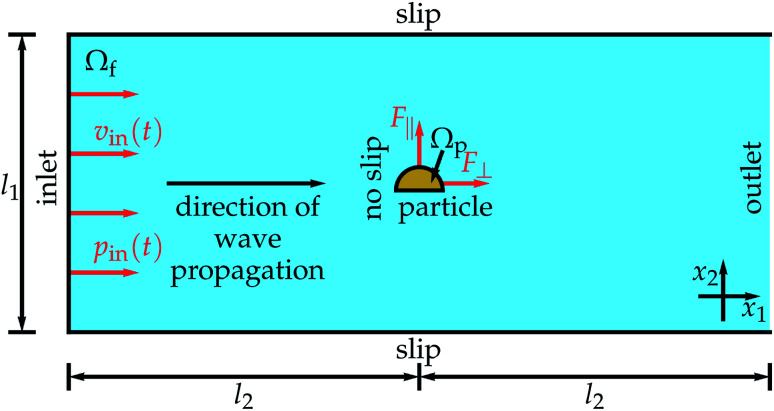
Setup for the simulations. A traveling ultrasound wave is entering the fluid domain *Ω*_f_ at the inlet, where it is prescribed by an inflow velocity *v*_in_(*t*) and pressure *p*_in_(*t*). The width of the fluid domain is *l*_1_ and the rigid particle, constituting a particle domain *Ω*_p_, is placed at a distance *l*_2_ from the inlet. At the particle boundary a no-slip condition is prescribed and for the lateral boundaries of *Ω*_f_ slip boundary conditions are used. The ultrasound exerts on the particle a time-averaged propulsion force with a component *F*_∥_ parallel to and a component *F*_⊥_ perpendicular to the particle orientation and after a further distance *l*_2_ the domain *Ω*_f_ ends with an outlet.

The setup for our study is shown in Fig. 1. A traveling ultrasound wave with frequency *f* = 1 MHz enters a rectangular domain *Ω*_f_ of an initially quiescent fluid, which we assume to be water, at an inlet of width *l*_1_ = 200*σ*, where *σ* = 1 μm is the particle diameter. At the inlet, the ultrasound wave is prescribed by a time-dependent inflow velocity *v*_in_(*t*) = (Δ*p*/(*ρ*_0_*c*_f_))sin(2π*ft*) perpendicular to the inlet and a time-dependent inflow pressure *p*_in_(*t*) = Δ*p* sin(2π*ft*) with the pressure amplitude Δ*p* = 10 kPa, the density of the quiescent fluid *ρ*_0_ = 998 kg m^−3^, and the sound velocity in the fluid *c*_f_ = 1484 m s^−1^. The pressure amplitude corresponds to an acoustic energy density *E* = Δ*p*^2^/(2*ρ*_0_*c*_f_^2^) = 22.7 mJ m^−3^. Starting at the inlet, the ultrasound wave propagates through the fluid domain parallel to its lateral boundaries, where we prescribe slip boundary conditions. After a distance *l*_2_ = *λ*/4 with the wavelength of the ultrasound *λ* = 1.484 mm, the wave arrives the fixed rigid particle, which is oriented perpendicular to the propagation direction of the wave. In the simulations, we describe the particle by a particle domain *Ω*_p_ and prescribe no-slip conditions at the particle's boundary ∂*Ω*_p_. Through the ultrasound, a time-averaged propulsion force with a component *F*_∥_ parallel to and a component *F*_⊥_ perpendicular to the particle's orientation is exerted on the particle. The wave then propagates a further distance *l*_2_ until it reaches an outlet at the end of the domain *Ω*_f_.

To avoid approximations like perturbation expansions that are involved in all previous studies using analytical^45,46^ or numerical^41,42,44^ methods to determine the propulsion velocity of acoustically propelled particles, we base our work on direct fluid dynamics simulations. Our simulations are carried out by numerically solving the compressible Navier–Stokes equations together with the continuity equation for the mass-density field *ρ*(*x⃑*,*t*) of the fluid and a constitutive equation for the fluid's pressure field *p*(*x⃑*,*t*) in the two-dimensional fluid domain *Ω*_f_. Here, *x⃑* = (*x*_1_,*x*_2_)^T^ is the position vector and *t* denotes time.

When *v⃑*(*x⃑*,*t*) with *v⃑* = (*v*_1_,*v*_2_)^T^ is the velocity field of the fluid and we use the short notation *∂*_*i*_ = *∂*/*∂*_*x_i_*_ with *i* ∈ {1, 2} for the spatial derivatives, the continuity equation that describes mass conservation is given by^57^1
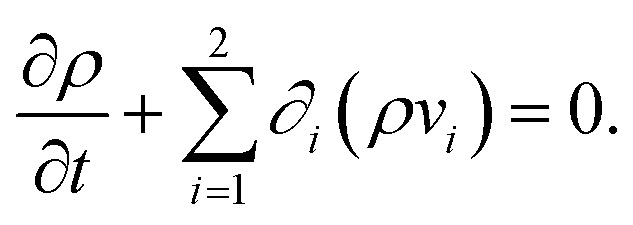


Momentum conservation of the fluid is then described by the Navier–Stokes equations^57^2
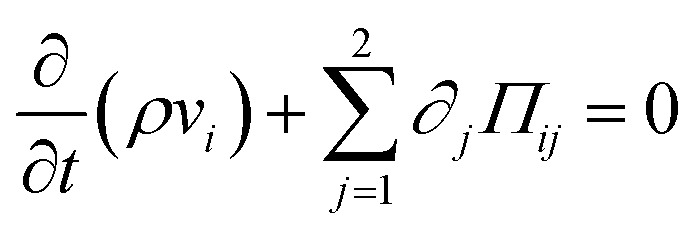
with the momentum-current tensor

3 *Π*_*ij*_ = *ρv*_*i*_*v*_*j*_ − *Σ*_*ij*_

and the stress tensor

4 *Σ*_*ij*_ = *Σ*_*ij*_^(*p*)^ + *Σ*_*ij*_^(*v*)^

for *i*, *j* ∈ {1, 2}. The stress tensor consists of the pressure part

5 *Σ*_*ij*_^(*p*)^ = −*pδ*_*ij*_

and viscous part6
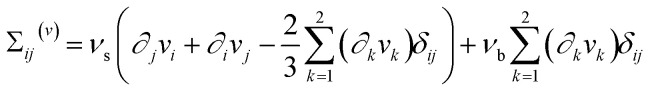
with the Kronecker symbol *δ*_*ij*_, shear viscosity (also called “dynamic viscosity”) *ν*_s_, and bulk viscosity (also called “volume viscosity”) *ν*_b_. Heat conduction and heating of the fluid by the sound waves are neglected here, since the ultrasound intensities that are used in experiments with acoustically propelled colloidal particles are usually rather small. To close the set of eqn (1)–(6), we need a constitutive equation for the pressure *p*(*x⃑*,*t*). When the sound intensity is sufficiently small, so that the fluid is acoustically nondispersive and heating of the fluid by the sound wave can be neglected, the local pressure *p*(*x⃑*,*t*) is given by the constitutive equation

7 *p*(*ρ*) = *p*_0_ + *c*_f_^2^(*ρ* − *ρ*_0_)

as a function of the local mass density *ρ*(*x⃑*,*t*). Here, *p*_0_ = *p*(*ρ*_0_) is the constant mean pressure of the fluid. To solve eqn (1)–(7) numerically, we used the software package OpenFOAM,^58^ which applies the finite volume method.

The time-dependent force *F⃑*(*t*) with *F⃑* = (*F*_1_,*F*_2_)^T^ and the time-dependent torque *T*(*t*) acting on the particle are calculated in the laboratory frame. Since the particle, which is described by the particle domain *Ω*_p_, has no-slip boundary conditions and is fixed in space in our simulations, the fluid velocity *v⃑*(*x⃑*,*t*) is zero at the fluid–particle interface ∂*Ω*_p_. So the total force and torque exerted on the particle are given by *F⃑* = *F⃑*^(*p*)^ + *F⃑*^(*v*)^ and *T* = *T*^(*p*)^ + *T*^(*v*)^ with the components^57^8
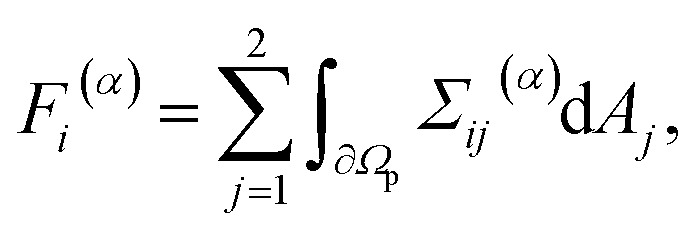
9
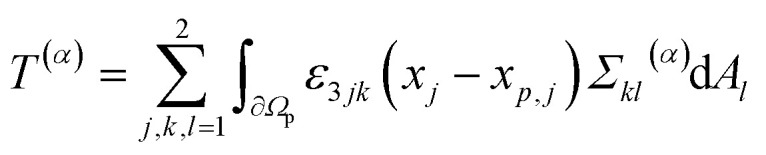
for *α* ∈ {*p*, *v*}. Here, d*A⃑*(*x⃑*) with d*A⃑* = (d*A*_1_,d*A*_2_)^T^ is the normal and outwards oriented surface element of ∂*Ω*_p_ at position *x⃑* = (*x*_1_,*x*_2_)^T^ when *x⃑* ∈ ∂*Ω*_p_, *ε*_*ijk*_ the Levi-Civita symbol, and *x⃑*_p_ = (*x*_p,1_,*x*_p,2_)^T^ the center-of-mass position of the particle. Since the position of the particle is fixed in our simulations, the results for the propulsion force correspond to a particle that is held in place. For a freely moving particle, the results correspond to a particle material with infinite mass density and can thus be seen as an upper bound for the propulsion force of a particle made of gold or another material with a large mass density.

By time-averaging *F⃑*^(*p*)^(*t*), *F⃑*^(*v*)^(*t*), *T*^(*p*)^(*t*), and *T*^(*v*)^(*t*) locally over one period *τ* of the ultrasound wave in the stationary state (*i.e.*, for large *t*), we obtain the time-averaged stationary forces 〈*F⃑*^(*p*)^〉, 〈*F⃑*^(*v*)^〉, and 〈*F⃑*〉 = 〈*F⃑*^(*p*)^〉 + 〈*F⃑*^(*v*)^〉 and the time-averaged stationary torques 〈*T*^(*p*)^〉, 〈*T*^(*v*)^〉, and 〈*T*〉 = 〈*T*^(*p*)^〉 + 〈*T*^(*v*)^〉, where 〈·〉 denotes the time average. Using 〈*F⃑*〉, we then calculate the time-averaged stationary propulsion velocity^59,60^10
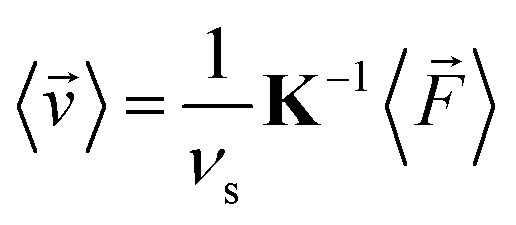
with the translational resistance matrix **K** of the considered particle. A similar equation can be used to calculate the time-averaged angular velocity11
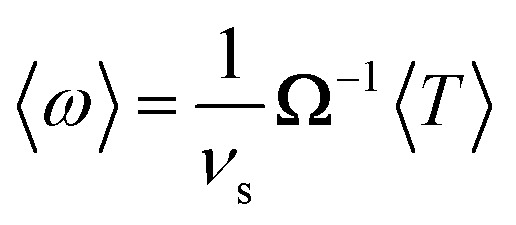
of the particle, where **Ω** is the particle's rotational resistance matrix. Both **K** and **Ω** are determined using the software HydResMat.^60,61^ The Stokes drag eqn (10) and (11) are valid for low Reynolds numbers Re ≪ 1 and have been frequently applied in similar cases in the literature.^1,33,34,39^ As we simulate a two-dimensional system to keep the computational effort manageable, but **K** and **Ω** are 3 × 3-dimensional matrices that correspond to a three-dimensional particle, we cannot apply eqn (8)–(11) directly. Therefore, we assign a thickness of *σ* to the particle, which equals its diameter, so that **K** and **Ω** can be calculated. Neglecting contributions by the lower and upper surfaces of the particle, we then use the three-dimensional versions of eqn (8)–(11). In principle, there is also a resistance matrix **C** that describes hydrodynamic translational–rotational coupling and has to be taken into account in eqn (10) and (11). However, since this coupling is small for the particle shapes considered in this work and would have only a negligible effect on our results, we neglect it here. The calculation of 〈*v⃑*〉 and 〈*ω*〉 thus involves three approximations. First, we use the flow field around the particle, which we have determined only for two instead of three spatial dimensions, to calculate the propulsion force and torque. Second, we use the time-averaged propulsion force and torque acting on the particle with fixed position and orientation to determine the time-averaged propulsion velocity and angular velocity such a particle would have if it could move freely. Third, we neglect hydrodynamic translational–rotational coupling associated with the particle shape.

From 〈*F⃑*〉 and 〈*v⃑*〉, we directly obtain the propulsion-force components *F*_∥_ = (〈*F⃑*〉)_2_ = *F*_∥,*p*_ + *F*_∥,*v*_ and *F*_⊥_ = (〈*F⃑*〉)_1_ = *F*_⊥,*p*_ + *F*_⊥,*v*_, their pressure components *F*_∥,*p*_ = (〈*F⃑*(*p*)〉)_2_ and *F*_⊥,*p*_ = (〈*F⃑*(*p*)〉)_1_ and viscous components *F*_∥,*v*_ = (〈*F⃑*(*v*)〉)_2_ and *F*_⊥,*v*_ = (〈*F⃑*(*v*)〉)_1_, as well as the propulsion-velocity components *v*_∥_ = (〈*v⃑*〉)_2_ and *v*_⊥_ = (〈*v⃑*〉)_1_ as the force- and velocity contributions parallel and perpendicular to the particle's orientation, *i.e.*, parallel and perpendicular to the *x*_2_ axis.

Nondimensionalization of the equations introduced above leads to the Helmholtz number He, a Reynolds number corresponding to the shear viscosity Re_s_, another Reynolds number corresponding to the bulk viscosity Re_b_, and the product Ma^2^Eu, with the Mach number Ma and Euler number Eu, corresponding to the pressure amplitude Δ*p* of the ultrasound wave that enters the simulated system. Table 1 shows the names, symbols, and assigned values of the parameters that are relevant for our simulations. The parameters related to the fluid are based on assuming that the fluid is water at normal temperature *T*_0_ = 293.15 K and normal pressure *p*_0_ = 101325 Pa. With the parameter values from Table 1, our simulations correspond to the following values of the dimensionless numbers:

12 He = 2π*fσ*/*c*_f_ ≈ 4.234 × 10^−3^,

13 Re_s_ = *ρ*_0_*c*_f_*σ*/*ν*_s_ ≈ 1478,

14 Re_b_ = *ρ*_0_*c*_f_*σ*/*ν*_b_ ≈ 516,

15 Ma^2^Eu = Δ*p*/(*ρ*_0_*c*_f_^2^) ≈ 4.550 × 10^−6^.

The Reynolds number 
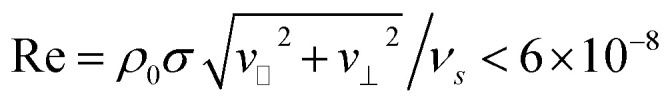
 characterizing the particle motion through the fluid is much less than one, when using the results for the propulsion velocity described in section III, and thus ensures that eqn (10) and (11) are applicable.

**Table 1:** Parameters that are relevant for our simulations and their values. We obtained the bulk viscosity *ν*_b_ for water at temperature *T*_0_ = 293.15 K by a cubic spline interpolation of the data from Table 1 in ref. 62

Name	Symbol	Value
Particle diameter	*σ*	1 μm
Sound frequency	*f*	1 MHz
Speed of sound	*c*_f_	1484 m s^−1^
Time period of sound	*τ* = 1/*f*	1 μs
Wavelength of sound	*λ* = *c*_f_/*f*	1.484 mm
Mean mass density of fluid	*ρ*_0_	998 kg m^−3^
Mean pressure of fluid	*p*_0_	101 325 Pa
Initial velocity of fluid	*v⃑*_0_	0⃑ m s^−1^
Sound pressure amplitude	Δ*p*	10 kPa
Acoustic energy density	*E* = Δ*p*^2^/(2*ρ*_0_*c*_f_^2^)	22.7 mJ m^−3^
Shear/dynamic viscosity of fluid	*ν*_s_	1.002 mPa s
Bulk/volume viscosity of fluid	*ν*_b_	2.87 mPa s
Domain width	*l*_1_	200*σ*
Inlet-particle or particle-outlet distance	*l*_2_	*λ*/4
Mesh-cell size	Δ*x*	15 nm to 1 μm
Time-step size	Δ*t*	1–10 ps
Simulation duration	*t*_max_	500*τ*

We discretized the fluid domain *Ω*_f_ using a structured mixed rectangular-triangular mesh with about 250 000 cells. The typical cell size Δ*x* varied from about 15 nm near the particle to about 1 μm far away from the particle. For the time integration, we used an adaptive time-step method with a maximum time-step size ensuring that the Courant–Friedrichs–Lewy number16
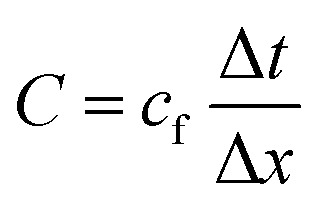
is smaller than one. The typical time-step size Δ*t* was thus between 1 ps and 10 ps. The simulations ran for *t*_max_ = 500*τ* to get sufficiently close to the stationary state. Due to the fine discretization in space and time and the relatively large spatial and temporal domains, the simulations were computationally very expensive and required a strong parallelization. The typical duration of one simulation was about 36 000 CPU core hours.

Since the simulations would require even more time to fully converge, we determined the stationary forces *F*_∥,*p*_, *F*_∥,*v*_, *F*_⊥,*p*_, and *F*_⊥,*v*_ and the stationary torque 〈*T*〉 by extrapolation. For this purpose, we used the fit function17
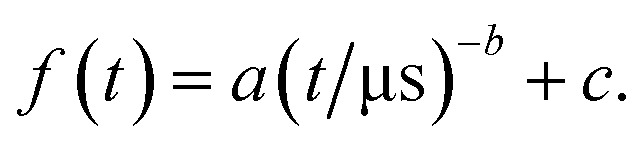


**Fig. 2:**
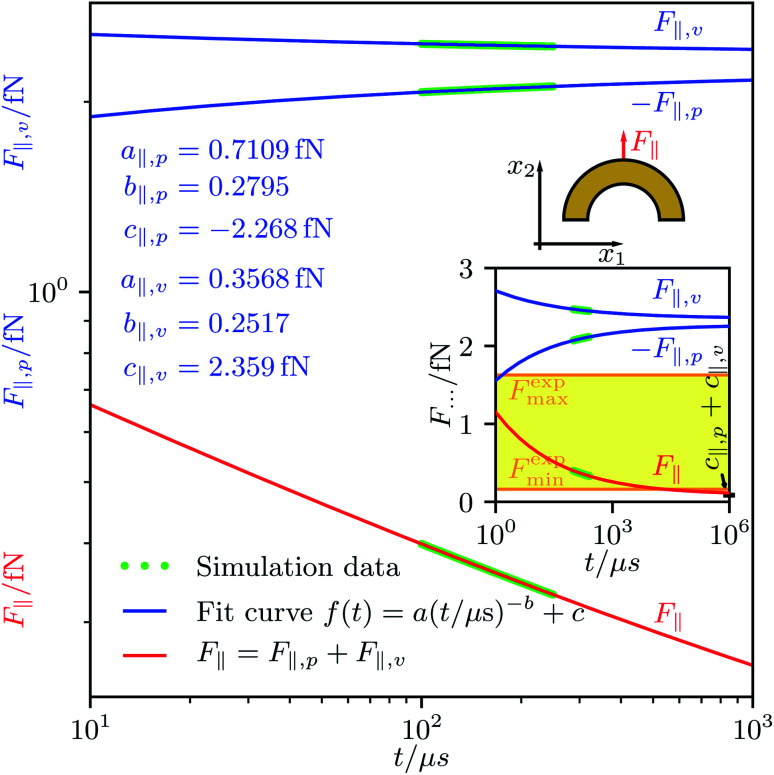
Simulation data for the time-dependent forces *F*_∥,*p*_(*t*) and *F*_∥,*v*_(*t*) acting on a particle with the shape of a hollow half ball as well as an extrapolation of the forces with the fit function *f*(*t*). The extrapolation of the total propulsion force parallel to the particle orientation *F*_∥_(*t*) = *F*_∥,*p*_(*t*) + *F*_∥,*v*_(*t*) converges against *c*_∥,*p*_ + *c*_∥,*v*_, where *c*_∥,*p*_ and *c*_∥,*v*_ are the offset fit coefficients in *f*(*t*) for *F*_∥,*p*_(*t*) and *F*_∥,*v*_(*t*), respectively. Its limiting value is consistent with corresponding experimental data from Soto *et al.*^38^ that can be tied to the interval [*F*^exp^_min_, *F*^exp^_max_] = [0.163 fN, 1.63 fN].

An example for the extrapolation, corresponding to a hollow-half-ball particle and considering the forces *F*_∥,*p*_ and *F*_∥,*v*_, is shown in Fig. 2. The fit curves for the other forces, torques, and particle shapes look similar. In the inset of this figure, the experimental data^38^ available for the addressed particle shape are indicated by a yellow band. The values of the fit parameters for all considered particle shapes are listed in Table 2.

**Table 2:** Fit parameters of the function (17) for the force components *F*_∥,*p*_ (subscript “∥, *p*”), *F*_∥,*v*_ (subscript “∥, *v*”), *F*_⊥,*p*_ (subscript “⊥, *p*”), and *F*_⊥,*v*_ (subscript “⊥, *v*”) for each considered particle shape. The second-last column corresponds to Fig. 2

Fit parameter	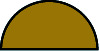 Half ball	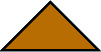 Cone	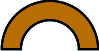 Hollow half ball	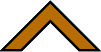 Hollow cone
*a*_∥,*p*_	0.5188 fN	0.7301 fN	0.7109 fN	0.9332 fN
*b*_∥,*p*_	0.2825	0.2919	0.2795	0.2808
*c*_∥,*p*_	−2.995 fN	−6.455 fN	−2.268 fN	−6.007 fN
*a*_∥,*v*_	0.2506 fN	0.2033 fN	0.3568 fN	0.2686 fN
*b*_∥,*v*_	0.2447	0.2180	0.2517	0.2104
*c*_∥,*v*_	3.073 fN	6.943 fN	2.359 fN	6.529 fN
*a*_⊥,*p*_	−0.4885 pN	−0.4563 pN	−0.5982 pN	−0.5250 pN
*b*_⊥,*p*_	1.240	1.234	1.240	1.233
*c*_⊥,*p*_	0.07838 fN	0.08258 fN	0.1011 fN	0.09072 fN
*a*_⊥,*v*_	−0.7505 pN	−0.6662 pN	−0.6360 pN	−0.5802 pN
*b*_⊥,*v*_	1.240	1.234	1.240	1.233
*c*_⊥,*v*_	0.1253 fN	0.1050 fN	0.1026 fN	0.09062 fN

## III. Results and discussion

**Fig. 3:**
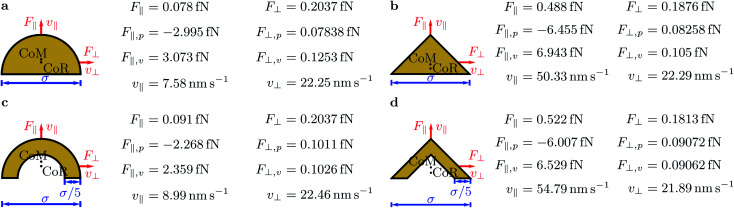
The considered particle shapes: (a) half ball, (b) cone, (c) hollow half ball, and (d) hollow cone. All particles have diameter *σ* and the hollow particles have wall width *σ*/5. The center of mass (CoM), the center of resistance (CoR), the direction of the propulsion-force component *F*_∥_ and corresponding propulsion-velocity component *v*_∥_, which are parallel to the symmetry axes of the particles and perpendicular to the main direction of sound propagation, and the direction of the components *F*_⊥_ and *v*_⊥_, which are perpendicular to the symmetry axes of the particles and parallel to the main direction of sound propagation, are indicated. For each particle shape, the values of *F*_∥_ = *F*_∥,*p*_ + *F*_∥,*v*_ and *F*_⊥_ = *F*_⊥,*p*_ + *F*_⊥,*v*_, the pressure components *F*_∥,*p*_ and *F*_⊥,*p*_, the viscous components *F*_∥,*v*_ and *F*_⊥,*v*_, and *v*_∥_ and *v*_⊥_ are given.

Since we are interested in studying the effect of pointedness and hollowness on the particle propulsion, we consider four different particle shapes: a half ball, a cone, and hollowed-out versions of both shapes (see Fig. 3).

All considered particles have diameter *σ* and the hollow ones have wall width *σ*/5. Both the particles' center of mass (CoM) and center of resistance (CoR) are on the symmetry axes of the particles. These particle shapes are chosen, since they are relatively simple with an axis of rotational symmetry, have a head-tail asymmetry that is necessary for acoustic propulsion, and differ with respect to pointedness and hollowness. The half ball is neither pointed nor hollow, the cone is pointed but not hollow, the hollow half ball is not pointed but hollow, and the hollow cone is both pointed and hollow. Due to the huge computational expense of the simulations, we did not consider additional particle shapes. A further motivation for choosing the mentioned particle shapes is the fact that there exist experimental data from a previous study that considered cup-shaped particles that are similar to our hollow half ball.^38^

Fig. 3 shows also the results for the propulsion force parallel to the particles' symmetry axis *F*_∥_ = *F*_∥,*p*_ + *F*_∥,*v*_, the propulsion force perpendicular to this axis *F*_⊥_ = *F*_⊥,*p*_ + *F*_⊥,*v*_, their pressure components *F*_∥,*p*_ and *F*_⊥,*p*_ and viscous components *F*_∥,*v*_ and *F*_⊥,*v*_, and the corresponding propulsion-velocity components *v*_∥_ and *v*_⊥_. For all particle shapes, *F*_∥_ and *v*_∥_ are positive. The components *F*_∥,*p*_ and *F*_∥,*v*_ are always negative and positive, respectively, where the latter component is dominating. Remarkably, both cone-shaped particles are associated with propulsion speeds *v*_∥_ ≈ 50 nm s^−1^ that are one order of magnitude larger than those for the half-ball shapes. This suggests that pointed shapes allow much faster acoustic propulsion parallel to the symmetry axis than rounded ones. Comparing the corresponding filled and hollowed-out particle shapes reveals that the hollow particles reach slightly (less than 20 percent) larger propulsion speeds parallel to the symmetry axis than their filled counterparts. This suggests that cavities in the particles have no significant effect on their propulsion speed. Among the considered particles, that with a hollow-cone shape reaches the largest propulsion force *F*_∥_ = 0.522 fN and the largest propulsion speed *v*_∥_ = 54.79 nm s^−1^.

Our result for *v*_∥_ for the hollow-half-ball particle can be compared to results from experiments described in ref. 38, where particles with a similar shape and size were found to propel with speed *v*_∥_ = 82.4(54) μm s^−1^. However, the comparison is complicated by the fact that this reference mentions not the acoustic energy density the particles were exposed to, but instead, as it is usual in experimental studies on acoustically propelled particles, only the amplitude of the alternating voltage applied to the piezoelectric transducer. To estimate the energy density that is related to the known voltage amplitude, we use the typical energy-density values for some voltage ranges given in ref. 55. According to this reference, a voltage amplitude lower than 10 V, as is used in the experiments described in ref. 38, is typically associated with an acoustic energy density of 10–100 J m^−3^. In our simulations, the energy density was 22.7 mJ m^−3^ and thus much smaller than in the experiments. Calculating the propulsion force *F*_∥_ that corresponds to the propulsion speed *v*_∥_ reported in the experiments and assuming that this force scales linearly with the acoustic energy density, we find a range of force values [*F*^exp^_min_, *F*^exp^_max_] with *F*^exp^_min_ = 0.163 fN and *F*^exp^_max_ = 1.63 fN that could have been observed in the experiments when using the same energy density as in our simulations. This is consistent with our finding for *F*_∥_. To be precise, our value for *F*_∥_ is slightly below *F*^exp^_min_, but given that *F*^exp^_min_ and *F*^exp^_max_ have been determined by a rough estimate, that we simulated traveling ultrasound waves whereas the experiments involved standing waves, that the frequency of the ultrasound was different in the simulations and experiments, and that we applied additional approximations as described in section II, the agreement of the force interval estimated from the experimental data with our result is very good.

The force components *F*_⊥_, *F*_⊥,*p*_, and *F*_⊥,*v*_ are all positive. This is not trivial, since these force components are a result of two opposing physical effects: the acoustic radiation force, which originates from the scattering of the ultrasound wave by the particles, and the acoustic streaming force, which originates from acoustic streaming caused by viscous damping of the ultrasound in the fluid and the associated drag on the particles. While the acoustic radiation force points in the direction of propagation of the ultrasound, the acoustic streaming velocity near the particles points in the opposite direction. For the particles and parameter values considered in this work, the acoustic radiation force is always dominant, but this can change for other particle sizes and shapes. When considering a sphere in a standing sound wave with frequency 1 MHz, the critical radius at which the acoustic radiation force and the acoustic streaming force balance each other is about 1 μm.^55^ For nonspherical particles and traveling waves, the critical radius is different. The force components *F*_⊥_, *F*_⊥,*p*_, and *F*_⊥,*v*_ and the velocity component *v*_⊥_ are rather similar for all considered particle shapes. In all cases, we found *F*_⊥_ ≈ 0.2 fN and *v*_⊥_ ≈ 22 nm s^−1^. For the half-sphere-shaped particles, *F*_⊥_ is 2–3 times larger than *F*_∥_. In contrast, for cone-shaped particles, *F*_⊥_ is 2–3 times smaller than *F*_∥_, which makes these particles more relevant as active colloidal particles.

We found no significant torques acting on the particles. This suggests that the particle orientation considered in this study is stable.

**Fig. 4:**
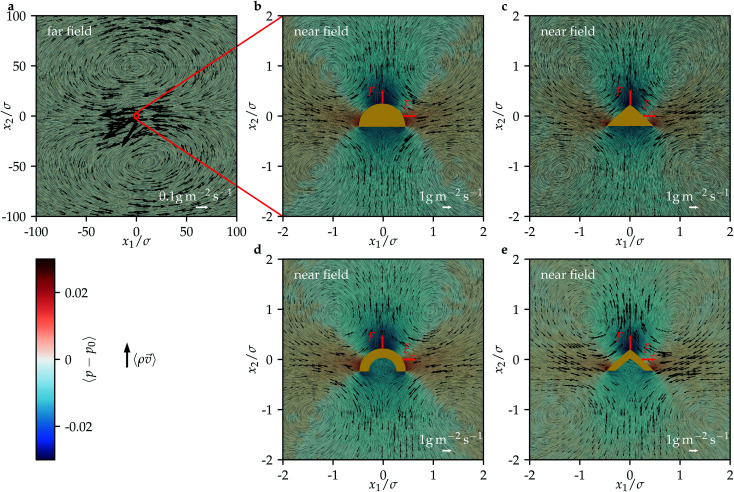
The time-averaged mass-current density 〈*ρv⃑*〉 and reduced pressure 〈*p* − *p*_0_〉 for all considered particle shapes (see Fig. 3). (a) The far field is shown for a half-ball particle and looks similar for the other particle shapes; (b)–(e) the near field is shown for each particle shape.

Next, we study the flow field around the particles. Fig. 4 shows the time-averaged mass-current density 〈*ρv⃑*〉 and reduced pressure 〈*p* − *p*_0_〉 for all considered particles. In each case, the far field of the flow shows two large counter-rotating vortices with diameters of about 100*σ*, which are in front of and behind the particle, respectively. Such a flow field is typical for acoustic streaming around a particle exposed to ultrasound even for simple shapes like a sphere.^63^ The flow from the far field seems to move the particle in the direction antiparallel to the propagation of the ultrasound, but in fact there acts also the acoustic radiation force on the particle, which is oriented parallel to the propagation direction and here dominates the acoustic streaming force that is exerted by this flow. Also the near field of the flow is similar for all considered particle shapes. The structure of the near field, however, is qualitatively different from that of the far field. There are now four vortices to the left and right in front of and behind the particles. The diameter of these vortices is about 2*σ*. There is an inflow towards the particles from their left and right and an outflow away from the particles at their front and back. For the reduced pressure, negative values are observed in front of and behind the particles, whereas positive values are observed to their left and right. The near field looks rather symmetric, but this does not prevent propulsion of the particles. Since the head-tail symmetry of the particles is broken, even a fully symmetric near field could lead to propulsion.

The structure of the near fields is similar to that of the flow fields generated by pusher squirmers.^64,65^ Identifying the particles as pushers would be an important result, since it would allow us to translate the known properties and modeling techniques for pushers to the so far much less investigated acoustically propelled particles. However, based on the present simulations with flow fields in two spatial dimensions and the consideration only of particles that are oriented perpendicular to the propagation direction of the ultrasound, it cannot be assessed whether the flow fields around ultrasound-propelled particles and pushers are in fact similar. Our results for the flow fields suggest that they are caused by local acoustic streaming close to the particles, as predicted in the theoretical work 45.

## IV. Conclusions

Based on direct numerical simulations, we have studied the acoustic propulsion of nonspherical nano- and microparticles by traveling ultrasound. For particle shapes that were either rounded or pointed and either filled or hollow, we have calculated the propulsion force acting on the particle, the resulting propulsion velocity, as well as the flow field around the particle. This allowed us to obtain important new insights into the propulsion of such particles. Our results, which are consistent with experiments from Soto *et al.*^38^ for cup-shaped particles, confirmed that the particles' propulsion parallel to their symmetry axis is very sensitive to the particle shape.^46^ The results revealed that a particle with a pointed shape can show a much more efficient propulsion than one with a rounded shape and that a cavity in the particle shape has no significant effect on the propulsion efficiency. For the propulsion perpendicular to the particles' symmetry axis, no significant dependence on the shape was found. Considering the small number of different particle shapes that have been addressed in previous studies, these findings suggest to use conical particles in future studies further investigating or applying acoustically propelled colloidal particles to reach higher propulsion speeds than for cup-shaped particles^38^ and the commonly used bullet-shaped particles.^1,33,34,39^ Since we found only a negligible effect of a cavity in the particle shape on its propulsion efficiency, the conical particle can have or have not a cavity, depending on which particle shape is easier to synthesize. Knowing that the propulsion efficiency of the particles can significantly be enhanced by choosing a more suitable particle shape is an important insight with respect to potential applications of this type of active particles, *e.g.*, in nanomedicine, where the particles need a large propulsion speed to withstand blood flow while the ultrasound intensity is limited to physiologically harmless values.^16,66,67^ When the particles shall be used for drug delivery in nanomedicine, a filled particle is advantageous, as its larger volume is associated with a larger capacity for transporting drugs.

The obtained time-averaged flow fields support the understanding of the particles' propulsion mechanism as originating basically from local acoustic streaming, as predicted by Nadal and Lauga.^45^ Remarkably, the flow's near field was observed to be similar to that of a pusher squirmer. A future study determining the full flow field in three spatial dimensions and for different particle orientations will have to clarify whether this agreement applies only to the particular situation studied here or these particles in fact constitute pushers. Identifying the particles as pushers would allow us to transfer the large existing knowledge about the motion of pushers and, *e.g.*, their interactions with obstacles and other particles to ultrasound-propelled particles, for which most of these issues have not yet been addressed. We expect that this would strongly boost the future theoretical investigation of ultrasound-propelled colloidal particles. For example, one could then use modified squirmer models to describe the motion of the particles on much larger time scales, which are no longer set by the high frequency of the ultrasound but instead by the rather small time-averaged flow velocities near the particles. This would reduce the computational effort to simulate the motion of the particles by several orders of magnitude and enable studies that were well-nigh impossible up to now. In addition, such modeling would even allow for the application of new analytical approaches, such as field theories derived *via*, *e.g.*, symmetry-based modeling,^68,69^ classical dynamical density functional theory,^70,71^ or the interaction-expansion method^72^ for describing the collective dynamics of many interacting particles on mesoscopic or macroscopic scales. Furthermore, the identification of the ultrasound-propelled particles as pushers would have intriguing consequences for future materials science. Since it is known from suspensions of bacteria that pushers can strongly reduce the viscosity of a liquid, one could then expect that it is possible to use suspensions of ultrasound-propelled particles to realize novel active materials with a viscosity that can be tuned *via* the ultrasound intensity from the normal positive viscosity of the suspension in the absence of ultrasound through to suprafluidity up to even negative viscosities.^73,74^

If such a future study shows instead that the particles are not squirmers, it still needs to be clarified whether they can be seen as nano- or microswimmers or constitute actuated particles. For swimmers, the particles' direction of motion has to rotate with the particles and needs to be independent of the direction of propagation of the ultrasound wave, whereas for actuated particles the energy source transmits also directional information to the particles. Apart from that, the basic understanding of the details of the particles' propulsion mechanism should further be extended by additional computational fluid dynamics simulations. As a task for the future, *e.g.*, the dependence of the propulsion efficiency on the aspect ratio of conical particles should be studied in detail. Examples for other parameters, whose influence on the particles' propulsion still needs to be studied, are their orientation relative to the direction of sound propagation, the viscosity of the fluid, and the frequency of the ultrasound. Furthermore, the simulations should be extended towards three spatial dimensions and freely movable particles.

## Conflicts of interest

There are no conflicts of interest to declare.

## Supplementary Material
